# Supplementary material for: Genomic Characterization of Partial Tandem Duplication Involving the KMT2A Gene in Adult Acute Myeloid Leukemia
Source: Cancers (Basel). 2024 Apr 26;16(9):1693. doi: 10.3390/cancers16091693 (PMC11082951; doi:10.3390/cancers16091693)
Supplement: Supplementary file 1 [file cancers-16-01693-s001.zip › Figure S1.pdf]

	DNA Transcription (N=39)											Epigenetic/Histone Modification (N=38)						FLT3 (N=27)		Splicing Factors (N=16)			RAS,RAF, PI3K/ AKT Pathways (N=11)				Others (N=10)				JAK/STAT Pathway (N=4)				DNA Repair (N=2)	
	KMT2A-PTD	RUNX1	WT1	BCOR	CEBPA	CUX1	ETV6	PHF6	GATA2	IKZF1	IRF1	ASXL1	DNMT3A	TET2	IDH1	IDH2	EZH2	FLT3-ITD	FLT3-TKD	U2AF1	SRSF2	SF3B1	PTPN11	NRAS	KRAS	BRAF	STAG2	CBL	SH2B3	NPM1	JAK2	CALR	CSF3R	JAK1	TP53	PPM1D
**Variant per Gene**	41	12	5	5	4	3	3	3	2	1	1	9	8	8	6	5	2	22	5	7	5	4	2	5	3	1	5	3	1	1	1	1	1	1	1	1
1	1																											1								
2	1																			1																
3	1			1																				1	1				1							
4	1					1			1													1					1						1			
5	1	1											1			1	1	1																		
6	1																	1	1	1																
7	1																																			
8	1	1		1														1		1				1				1								
9	1	2	2											1				1				2														
10	1														1																					
11	1					1							1		1									1												1
12	1							1							1																					
13	1		1											1			1	2																		
14	1															1		1			1					1										
15	1																			1			1													
16	1																			1			1													
17	1											1			1																					
18	1				1													1																		
19	1	1			1													2	1																	
20	1				1							1															1									
21	1						2						2	1				1																		
22	1			1																1																
23	1								1				1	1				2																		
24	1	1				1							1						1					1								1				
25	1															1								1												
26	1	1	1		1								1					2																		
27	1											1		2																						
28	1			1												1																		1		
29	1	1		1											1				2		1						1									
30	1	1											1	1				1																		
31	1											1																								
32	1																				1										1					
33	1											1						3												1						
34	1	1									1							2		1																
35	1						1			1																	1	1								
36	1		1					2																												
37	1	1										1			1			1			1						1									
38	1	1										1		1							1				1											
39	1											1				1									1											
40	1											1										1														
41	1																																		1	

Figure S1. Co-occurring variants identified in KMT2A-PTD positive cases by NGS. Note that cases 2, 3 and 35 were determined not to have a KMT2A-PTD by MLPA and/or OGM. Variants were classified using the AMP/CAP/ASCO somatic variant classification guidelines (Li et al., 2017, PMID: 27993330). Red: Tier-1 and Tier 2. Grey: Tier-3.
