# Supplementary material for: Genomic Characterization of Partial Tandem Duplication Involving the KMT2A Gene in Adult Acute Myeloid Leukemia
Source: Cancers (Basel). 2024 Apr 26;16(9):1693. doi: 10.3390/cancers16091693 (PMC11082951; doi:10.3390/cancers16091693)
Supplement: Supplementary file 1 [file cancers-16-01693-s001.zip › Table S1.pdf]

Table S1. Clinical features of KMT2A-PTD patients identified by heme-NGS.

Note that cases 2, 3 and 35 were determined not to have a KMT2A-PTD by MLPA and/or OGM. AML: Acute Myeloid Leukemia. t-AML: therapy related AML. AML-NOS: AML Not otherwise specified. AML-MR: AML myelodysplasia related. MDS-EB2: MDS with excess blast 2. BPDCN. Blastic plasmacytoid dendritic cell neoplasm.

Case	Age	Gender	Final Diagnosis	Blast	WBC (10^9/L)	Platelets (10^9/L)	Hemoglobin (g/L)
1	3	F	AML	n/a	n/a	n/a	n/a
2	67	M	t-AML	24%	1.8	17	74
3	40	M	AML-NOS	95%	37.9	72	122
4	55	M	AML-MR	19%	8.5	11	n/a
5	38	F	AML-MR	85%	137.6	56	95
6	74	M	AML	46%	6.1	61	71
7	68	M	AML-MR	72%	119.5	20	86
8	62	M	AML-MR	40%	n/a	n/a	n/a
9	88	M	AML-MR	49%	76.4	73	86
10	82	M	AML-NOS	92%	18.5	38	81
11	22	M	AML-NOS	60%	27.9	110	94
12	70	M	AML-MR	90%	n/a	115	80
13	77	M	t-AML	57%	7.2	35	92
14	78	M	AML-MR	80%	81.9	134	128
15	88	M	AML-MR	83%	n/a	n/a	n/a
16	68	F	AML-MR	30%	2.7	3	64
17	62	M	AM-MR	90%	1.8	11	69
18	85	M	AML-NOS	65%	n/a	n/a	n/a
19	45	F	AML-MR	85%	51.6	16	91
20	59	M	AML-NOS	59%	3.6	122	105
21	53	M	AML-NOS	75%	4.9	12	70
22	70	M	AML-MR	25%	1.1	177	87
23	77	F	AML-NOS	14%	46.9	50	70
24	84	M	AML-NOS	21%	29.1	44	93
25	71	M	AML	40%	n/a	n/a	n/a
26	32	F	AML-MR	79%	n/a	n/a	n/a
27	86	M	MDS-EB2	16%	n/a	49	111
28	79	M	AML-MR	63%	n/a	n/a	n/a
29	76	M	AML-MR	65%	12.5	60	72
30	81	F	AML-MR	70%	146.4	91	92
31	82	M	MPAL	n/a	n/a	n/a	n/a
32	76	M	Myelofibrosis	5%	34.5	318	95
33	43	M	NPM1 mutated AML	47%	45	66	117
34	79	M	AML-MR	60%	245.3	71	64
35	25	F	AML	90%	37.6	43	109
36	65	M	AML	29%	n/a	n/a	n/a
37	64	M	AML-MR	20%	17	107	87
38	74	M	AML-MR	40%	83.2	33	93
39	81	F	BPDCN	10%	10.5	222	145
40	73	F	AML-MR	23%	n/a	n/a	n/a
41	64	M	AML-MR	36%	6	21	79
